# Circulating MicroRNAs as Novel Potential Biomarkers for Left Ventricular Remodeling in Postinfarction Heart Failure

**DOI:** 10.1155/2019/5093803

**Published:** 2019-12-02

**Authors:** Guangyuan Gao, Weiwei Chen, Miao Liu, Xu Yan, Ping Yang

**Affiliations:** ^1^Department of Cardiology, China-Japan Union Hospital of Jilin University, Changchun 130031, China; ^2^Jilin Provincial Molecular Biology Research Center for Precision Medicine of Major Cardiovascular Disease, Changchun 130031, China

## Abstract

Circulating microRNAs (miRNAs) have been proposed as potential biomarkers for left ventricular remodeling in postinfarction heart failure (HF). However, the diagnostic reproducibility of the use of circulating miRNAs may be affected by the temporal expression of miRNAs following myocardial infarction (MI). In the current study, using a MI-induced HF rat cohort (4-, 8-, and 12-week post-MI groups), we investigated the temporal expression of plasma miRNAs during the development of left ventricular remodeling. The plasma miRNA expression profile was obtained using miRNA sequencing. The expression of candidate miRNAs in plasma and tissues was examined with real-time PCR. Target genes of candidate miRNAs were predicted using a parallel miRNA-messenger RNA expression profiling approach. The value of plasma miRNAs as biomarkers for left ventricular remodeling was evaluated in patients with postinfarction HF (*n* = 32) and control patients with stable angina and without significant coronary lesions and HF (*n* = 16) with real-time PCR. Although the expression levels of miR-20a-5p, miR-340-5p, and let-7i-5p were temporally regulated in plasma, myocardium, and peripheral blood mononuclear cells, the expression levels of plasma miRNAs, especially miR-20a-5p, were associated with the development of left ventricular remodeling in the postinfarction HF rat cohort. The target genes of these 3 miRNAs were associated with the mechanistic target of rapamycin, nuclear factor-*κ*B, tumour necrosis factor, apoptosis, and p53 signaling pathways. Additionally, the plasma levels of miR-20a-5p, miR-340-5p, and let-7i-5p were significantly increased in patients with postinfarction HF. However, only the expression levels of miR-20a-5p presented significant positive correlations with left ventricular internal end diastolic dimension and left ventricular end diastolic volume. In conclusion, the expression levels of plasma miR-20a-5p were significantly associated with the degree of left ventricular dilatation, and plasma miR-20a-5p may be a potential biomarker for postinfarction left ventricular remodeling.

## 1. Introduction

Heart failure (HF) is one of the leading causes of hospital admission and mortality worldwide [1]. Myocardial infarction (MI) is a common predisposing cause of HF [1]. Following MI, progressive left ventricular remodeling occurs in the noninfarcted myocardium and plays a central role in the pathophysiology of HF [2, 3]. Left ventricular remodeling is characterized by left ventricular dilatation, hypertrophy, distortion of contour, activation of interstitial fibrosis, and deterioration in cardiac dysfunction [2–4]. In clinical practice, circulating biomarkers such as natriuretic peptides associated with cardiac overload facilitate the clinical management of HF [1]. Nevertheless, there is a need to find reliable circulating biomarkers for tracking the process of left ventricular remodeling to help clinicians recognize high-risk patients for more aggressive prevention as early as possible.

Recently, circulating microRNAs (miRNAs) have become an emerging class of biomarkers. miRNAs are a large class of small noncoding RNAs that are powerful posttranscriptional regulators of gene expression [5]. Gene expression reprogramming is the base of ventricular remodeling [6]. Several studies have demonstrated that miRNAs are involved in the development of HF by silencing the expression of genes [7–9]. As miRNAs can be packaged in microparticles (exosomes, microvesicles, and apoptotic bodies) or bound to transport proteins, they are protected from degradation and can be detected by quantitative methods in the plasma samples [10]. Recent clinical studies have indicated that circulating miRNA may be valuable biomarkers for postinfarction left ventricular remodeling [11–18]. However, the diagnostic reproducibility of using circulating miRNAs as biomarkers for postinfarction cardiac remodeling remains imperfect, which may be due to their time-dependent expression during the development of HF, the complex mechanisms underlying the progression of cardiac remodeling, and the use of antiremodeling medical therapy [11–18].

The rat coronary artery ligation model exhibits many pathophysiologic and clinical characteristics that are similar to MI-induced HF in humans [19]. Progressive left ventricular dilatation can be continuously observed for up to 3-4 months following coronary artery ligation [19]. In the present study, using a rat cohort (4-, 8-, and 12-week postcoronary artery ligation groups), we assessed the temporal expression of plasma miRNAs during the development of left ventricular remodeling and conducted a preliminary study to explore the possible origins and target genes of candidate miRNAs. The value of candidate plasma miRNAs as biomarkers for postinfarction left ventricular remodeling was subsequently examined in a case control study between patients with postinfarction HF and patients with stable angina (without significant coronary lesions and HF).

## 2. Materials and Methods

### 2.1. Animals

All animal procedures were conducted in accordance with the institutional guidelines for the care and use of laboratory animals at Jilin University, Jilin, China. All experimental procedures were approved by the Ethical Review Board of China-Japan Union Hospital of Jilin University. Male Wistar rats (8 weeks, weighing 250~280 g) were obtained from the Center for Laboratory Animals, Medical College, Jilin University, Changchun, China. The animals were subjected to sham surgery or surgery involving ligation of the left anterior descending artery. Briefly, the rats were anaesthetized with oxygen containing 3% isoflurane supplied by a rodent respirator. Following anaesthetization, the thorax was opened in the left parasternal area, and MI was induced by ligating the left anterior descending coronary artery using 3-0 suture between the pulmonary cone and left atrium. After 4 weeks, successful induction of HF was confirmed by echocardiography, and the animals were randomly divided into 4-week (*n* = 16), 8-week (*n* = 20), and 12-week (*n* = 16) post-MI groups and corresponding sham groups (*n* = 12, *n* = 16, and *n* = 12). At 4-week, 8-week, and 12-week post-MI induction, echocardiography was recorded. Animals were then euthanized, and the samples (heart, liver, spleen, lung, kidney, thymus, and blood) were quickly harvested. Tissue samples were conserved in liquid nitrogen until use. Blood was collected in ethylenediaminetetraacetic acid-treated tubes. Following preliminary centrifugation (2,000 × g, 10 minutes, 4°C), the plasma samples were further centrifuged (16,000 × g, 10 minutes, 4°C) to pellet platelets and cellular debris, and the aliquots of supernatant were stored at -80°C in a freezer. Peripheral blood mononuclear cells (PBMCs) were purified with Ficoll separating solution (TBDscience, Tianjin, China).

### 2.2. Patient Population

Patients with postinfarction HF admitted to China-Japan Union Hospital of Jilin University between December 2018 and June 2019 were eligible for the current study. Prior MI and HF were defined according to the current definitions [1, 20, 21]. Patients who met the criteria “pathological Q waves, in the absence of nonischemic causes and/or imaging evidence of the loss of viable myocardium in a pattern consistent with ischemic etiology” were diagnosed as prior MI [20]. Patients with symptoms and/or signs of HF, N-terminal pro-B type natriuretic peptide (NT − proBNP) > 125 pg/ml, and other cardiac functional and structural alterations underlying HF were diagnosed as HF [1, 21]. Considering that the expression levels of plasma miRNAs may be affected by atherosclerosis, hypertension, diabetes, and hyperlipaemia [10], patients with stable angina and without significant coronary lesions requiring percutaneous coronary intervention (>50% stenosis determined by angiography) [22] and HF, who often have similar risk factor baselines for coronary artery disease as patients with prior MI, were enrolled as controls. Demographic, clinical, and echocardiographic characteristics were abstracted from the electronic medical records. Patients were excluded from the current study when they were known to have a recent acute MI, autoimmune disease, infection, cardiogenic shock, valvular disease, severe renal insufficiency (eGFR < 30 ml/min/1.73 m^2^), malignancy, or pregnancy. Baseline characteristics were compared via chi-squared test (categorical variables), two-independent-sample *t*-test (normally distributed variables), or Mann-Whitney *U* test (nonnormally distributed variables). The present study was approved by the Ethical Review Board of the China-Japan Union Hospital of Jilin University, China. Plasma samples were obtained with informed consent for the proper secondary use of human samples at Jilin Provincial Molecular Biology Research Center for Precision Medicine of Major Cardiovascular Disease. All procedures, including blood storage and data collection were performed in accordance with the institutional guidelines for the use of human samples by China-Japan Union Hospital of Jilin University.

### 2.3. Echocardiography

Rats were mildly anaesthetized using 3% isoflurane, and transthoracic echocardiography was performed using a Vivid-i echocardiography machine (General Electric Company, Fairfield, CT, USA) equipped with an 11.5 MHz transducer. Electrocardiographic assessments for patients were performed by three cardiologists who were blinded to the clinical data.

### 2.4. Pathology

Histologic studies were conducted with 4% paraformaldehyde-fixed and paraffin-embedded left ventricular samples from rats of all groups. Haematoxylin/eosin staining and Masson's trichrome staining were performed on cross sections of the left ventricle. Images were acquired with a microscope (Olympus Corporation, Tokyo, Japan) equipped with a digital camera and cellSens Dimension software (version 1.16; Olympus Corporation, Tokyo, Japan).

### 2.5. ELISA Assay for BNP

The plasma levels of BNP in rats were measured by an ELISA assay (R&D Systems, Inc., Minneapolis, MN, USA) following the manufacturer's instructions. The optical density values of samples were detected by a spectrophotometer with a wavelength of 450 nm. The normalization curve was obtained from a series of BNP standards provided in the kit.

### 2.6. RNA Extraction

Total plasma RNA extraction was performed using TRIzol LS solution (Invitrogen; Thermo Fisher Scientific, Inc., Waltham, MA, USA) with a *Caenorhabditis elegans* (*C. elegans*) RNA template as a spike-in control miRNA for normalization. Total RNA from tissues and PBMCs of rats was extracted using a TRIzol solution (Invitrogen; Thermo Fisher Scientific, Inc.).

### 2.7. RNA Sequencing and Bioinformatic Analysis

Following the RNA integrity and concentration examination, 2.5 ng of RNA per plasma sample and 1 *μ*g of RNA per myocardial sample were used to prepare the library for small RNAs and messenger RNAs (mRNAs), respectively. Differential expression analysis for miRNAs or mRNAs between the 8-week post-MI group and the sham group was conducted using the DESeq package (version 1.10.1). miRNAs or mRNAs with a fold change (FC) < 0.5 or >2 were determined to be differentially expressed. The target genes of miRNAs were predicted using miRWalk (version 3.0), miRanda (version 3.3a), RNAhybrid (version 2.1.1), and TargetScan (version 6.2) analyses. The functions of the target genes were annotated using KOBAS (version 3.0) based on the Kyoto Encyclopedia of Genes and Genomes (KEGG) and the Gene Ontology (GO) databases.

### 2.8. Real-Time PCR

An aliquot of 3.8 *μ*l of RNA was reverse-transcribed to cDNA in a 10 *μ*l reaction using a Mir-X First-Strand Synthesis Kit (Takara Bio; Clontech Laboratories, Inc., Mountain View, CA, USA). Both miRNAs and mRNAs were polyadenylated, reverse-transcribed, and quantified using a Mir-X RT-qPCR kit (Takara Bio; Clontech Laboratories, Inc.). The expression levels of miRNAs were normalized to the expression levels of *C. elegans* miRNA-39 for plasma samples and U6 for tissue samples. The expression levels of mRNAs were normalized to the expression levels of *α*-actin. Specific primers for quantitative PCR were used as follows: DNA-damage-inducible transcript 4 (*Ddit4*) forward primer, 5′-GCTCTGGACCCCAGTCTAGT-3′; *Ddit4* reverse primer, 5′-GGGACAGTCCTTCAGTCCTT-3′; sestrin 1 (*Sesn1*) forward primer, 5′-AAGTGAGGTGGGAGGGTCTGTG-3′; *Sesn1* reverse primer, 5′-TTTGAAAGCCCGTCCGAAGGTC-3′; NF-*κ*B inhibitor *α* (*Nfkbia*) forward primer, 5′-GTCTGAACTCGCCACCCAACTG-3′; *Nfkbia* reverse primer, 5′-GTCCACCAACCGCTCCTTCTTG-3′; *α*-actin forward primer, 5′-GGGTATGGGTCAGAAGGACT-3′; and *α*-actin reverse primer, 5′-GAGGCATACAGGGACAACAC-3′. The entire sequence of mature miRNA with all U's converted to T's was used as a forward primer for each miRNA. The reverse primer was provided in the kit and was complementary to the adapter sequence of the reverse-transcribed primer.

### 2.9. Exosome Isolation

The isolation of plasma exosomes was supported by a Total Exosome Isolation Kit (Invitrogen; Thermo Fisher Scientific, Inc.) following the manufacturer's instructions. Proteinase K was used to remove the bulk of protein from the plasma. The exosome-containing pellet was resuspended in PBS. The morphology of the exosomes was observed by transmission electron microscopy. The exosomal markers were examined by western blot analysis. Exosome samples were homogenized in ice-cold RIPA buffer (consisting of 50 mM Tris (pH 7.4), 150 mM NaCl, 1% NP-40, 0.5% sodium deoxycholate, and 0.1% sodium dodecyl sulfate) supplemented with protease inhibitors (Beyotime Biotechnology, Shanghai, China). The total protein was loaded for resolution by sodium dodecyl sulfate-polyacrylamide gels under reducing conditions. The primary antibodies against apoptosis-linked gene 2-interacting protein X (ALIX; 1 : 2000; cat. no. ab186429), tumour susceptibility gene 101 protein (TSG101; 1 : 2000; cat. no. ab125011), and cluster of differentiation 63 (CD63; 1 : 1000; cat. no. ab108950) were purchased from Abcam (Cambridge, MA, USA). The HRP-conjugated secondary antibody (1 : 5000; cat. no. BS13278) was purchased from Bioworld Technology, Inc. (Louis Park, MN, USA).

### 2.10. Statistical Analysis

All measurement data are expressed as the means ± S.E.M. Statistical analysis was performed with PASW Statistics 18 software (version 18.0.0; IBM, Inc., Armonk, NY, USA). Differences between groups were tested by a two-independent-sample *t*-test. Statistical graphs were generated using GraphPad Prism software (version 7.04; GraphPad Software, Inc., San Diego, CA, USA). Receiver operator characteristic (ROC) curves and the area under the curve (AUC) were calculated using MedCalc software (version 18.11; MEDCALC, Inc., Ostend, Belgium). For all analyses, a *P* value < 0.05 was considered significantly different.

## 3. Results

### 3.1. General Characteristics of Postinfarction HF Rats

**Figure 1:**
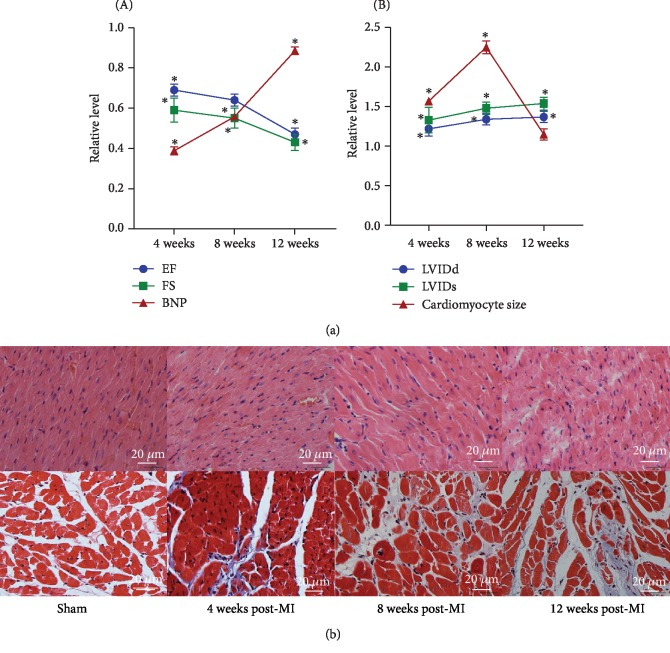
General characteristics of sham and post-MI rats. (a) Echocardiographic EF and FS measurements and plasma BNP levels of sham and post-MI rats at 4 weeks, 8 weeks, and 12 weeks post-MI (A). LVIDd, LVIDs, and cardiomyocyte size values of sham and post-MI rats at 4 weeks, 8 weeks, and 12 weeks post-MI (B). (b) Haematoxylin/eosin staining (upper) and Masson's trichrome staining (lower) of the left ventricle of sham and post-MI rats at 4 weeks, 8 weeks, and 12 weeks post-MI. ^∗^*P* < 0.05 versus the corresponding sham group. EF: ejection fraction; FS: fractional shortening; BNP: brain natriuretic peptide; MI: myocardial infarction; LVIDd: left ventricular internal end diastolic dimension; LVIDs: left ventricular internal end systolic dimension.

At 4 weeks post-MI, a reduction in the echocardiographic ejection fraction and fractional shortening measurements and an increase in the plasma levels of BNP were observed in post-MI rats (4-week post-MI group) compared with sham rats, indicating damage to cardiac function and the initiation of chronic HF (Figure 1(a)). Over time, cardiac function progressively deteriorated at 8 weeks post-MI (8-week post-MI group) and was dramatically damaged at 12 weeks post-MI (12-week post-MI group) (Figure 1(a)).

Echocardiographic left ventricular internal end diastolic dimension (LVIDd) and left ventricular internal end systolic dimension (LVIDs) values were significantly increased from 4 weeks post-MI to 8 weeks post-MI and mildly increased from 8 weeks post-MI to 12 weeks post-MI (Figure 1(a)). Progressively increased extracellular matrix synthesis and cardiac fibrosis, as determined by histopathology, were seen in the left ventricle of post-MI rats over time, which indicated the development of cardiac remodeling (Figure 1(b)). Furthermore, we found that the cardiomyocyte size in an area distant from the site of infarction was larger in the 4-week post-MI and 8-week post-MI groups than in the corresponding sham groups, and this cardiomyocyte hypertrophy was attenuated at 12 weeks post-MI (Figure 1(b)). The dilatation of the left ventricle, the activation of cardiac fibrosis, and cardiomyocyte hypertrophy may be compensatory mechanisms triggered to maintain cardiac function at 4 and 8 weeks post-MI. However, these mechanisms became maladaptive at 12 weeks post-MI and led to further left ventricular dilatation and thinning and significant deterioration in cardiac function.

### 3.2. Discovery of Candidate Plasma miRNAs

**Figure 2:**
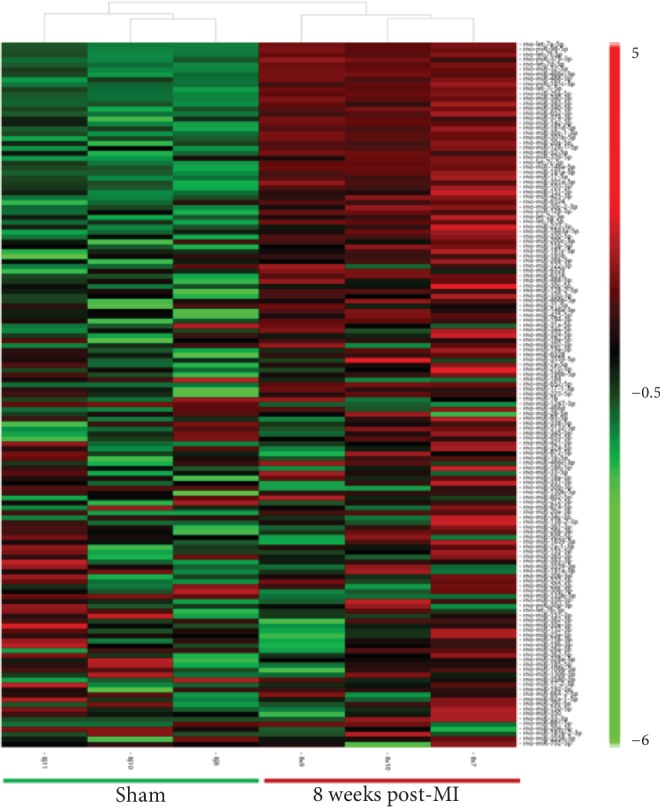
Hierarchical clustering of 143 DE circulating miRNAs (FC < 0.5 or >2; *P* < 0.05) in the 8-week post-MI group (*n* = 3) compared with the sham group (*n* = 3). DE: differentially expressed; FC: fold change.

We first analysed the global plasma miRNA expression in the 8-week post-MI group and corresponding sham group. We detected 619 known miRNAs in the plasma samples. Differential expression analysis indicated that 143 miRNAs were differentially expressed in the 8-week post-MI group compared with the sham group (FC < 0.5 or >2; *P* < 0.05). Of the 143 differentially expressed miRNAs, 138 were upregulated and 5 were downregulated in the 8-week post-MI group compared with the sham group. Hierarchical clustering analysis indicated that differentially expressed miRNAs could clearly distinguish the 8-week post-MI group from the sham group (Figure 2). From the 143 differentially expressed miRNAs, we selected 13 miRNAs based on their FCs and probability values for further study.

### 3.3. Validation of Candidate Plasma miRNAs

**Figure 3:**
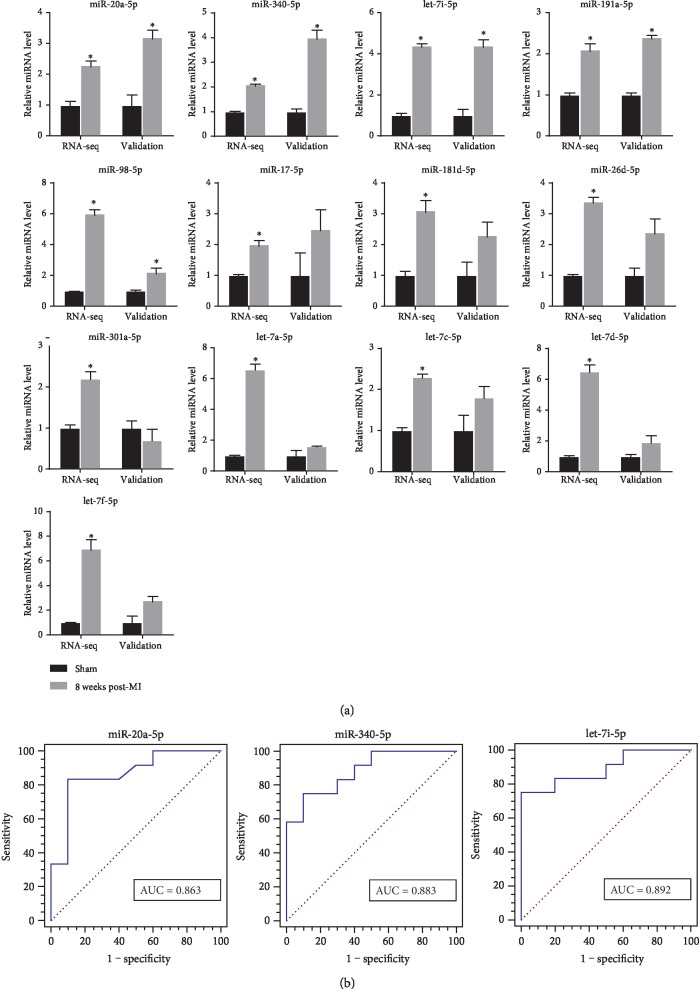
Validation of candidate plasma miRNAs. (a) Expression levels of 13 candidate plasma miRNAs in the 8-week post-MI group and the sham group. The left 2 bars of each panel show miRNA levels detected by miRNA sequencing in the 8-week post-MI group (*n* = 3) and the sham group (*n* = 3). The right 2 bars of each panel show the same miRNA levels validated using real-time PCR in the 8-week post-MI group (*n* = 16) and the sham group (*n* = 12). (b) ROC curves and the AUCs of miR-20a-5p, miR-340-5p, and let-7i-5p for predicting HF at 8 weeks post-MI. ^∗^*P* < 0.05 versus the corresponding sham group. RNA-seq: RNA sequencing; MI: myocardial infarction; HF: heart failure; ROC: receiver operator characteristic; AUC: area under the curve.

We validated the expression levels of 13 candidate miRNAs in the 8-week post-MI group and sham group. In the 8-week post-MI group, rats had an ejection fraction lower than 50%. In the sham group, rats had an ejection fraction higher than 70%. The FCs of the plasma miRNA levels in the 8-week post-MI group versus the sham group are shown in Figure 3(a). Of the 13 miRNAs that were significantly upregulated in miRNA sequencing, 5 miRNAs were also significantly upregulated in the validated 8-week post-MI group compared with the sham group. The expression levels of miR-20a-5p, miR-340-5p, and let-7i-5p were increased more than threefold in the 8-week post-MI group compared with the sham group. In addition, we evaluated the accuracy and sensitivity of miR-20a-5p, miR-340-5p, and let-7i-5p for predicting HF in the 8-week post-MI group and the sham group using a ROC curve and the AUC. The AUCs of miR-20a-5p, miR-340-5p and let-7i-5p were 0.863 (*P* < 0.05), 0.883 (*P* < 0.05), and 0.892 (*P* < 0.05), respectively (Figure 3(b)). The expression levels of miR-20a-5p, miR-340-5p, and let-7i-5p were closely associated with HF.

### 3.4. Temporal Expression of miRNAs in Plasma and Myocardium from 4 Weeks Post-MI to 12 Weeks Post-MI

**Figure 4:**
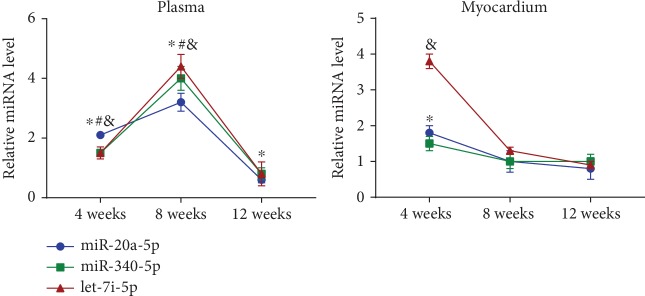
Temporal expression of miR-20a-5p, miR-340-5p, and let-7i-5p in the plasma and myocardium of post-MI rats from 4 weeks post-MI to 12 weeks post-MI. ^∗^*P* < 0.05 versus the corresponding sham group (miR-20a-5p); ^#^*P* < 0.05 versus the corresponding sham group (miR-340-5p); ^&^*P* < 0.05 versus the corresponding sham group (let-7i-5p). MI: myocardial infarction.

We evaluated the expression levels of miR-20a-5p, miR-340-5p, and let-7i-5p in plasma and myocardium of the post-MI rat cohort. The expression levels of miR-20a-5p, miR-340-5p, and let-7i-5p showed an upward trend from 4 weeks to 8 weeks post-MI, whereas their expression dramatically decreased to baseline levels at 12 weeks post-MI, and the expression levels of miR-20a-5p were significantly decreased in the 12-week post-MI group compared with the sham group (Figure 4). As shown in Table 1, there were significant positive correlations between the expression levels of miR-20a-5p, miR-340-5p, and let-7i-5p and LVIDd, LVIDs, and cardiomyocyte size at 4 weeks and 8 weeks post-MI. Additionally, inverse correlations were identified between the expression levels of miR-20a-5p and LVIDd and LVIDs at 12 weeks post-MI.

The expression levels of miR-20a-5p and let-7i-5p were elevated in the myocardium of the 4-week post-MI group compared with the sham group. No significant difference in the expression levels of miR-20a-5p, miR-340-5p, and let-7i-5p in the myocardium was identified between the post-MI groups and the sham groups at 8 and 12 weeks post-MI (Figure 4).

### 3.5. Possible Origins of Plasma miRNAs

**Figure 5:**
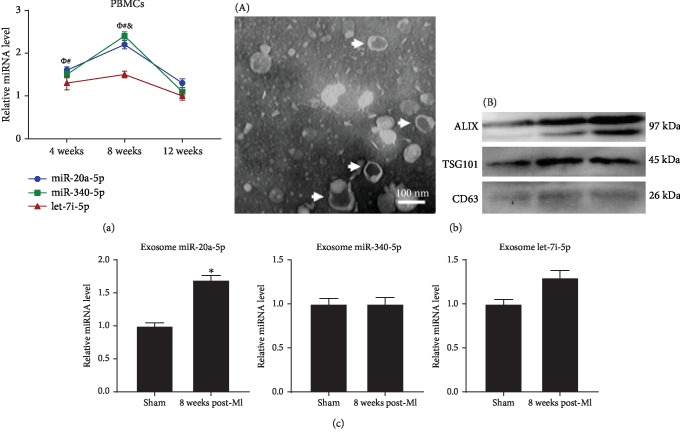
Possible origins of plasma miR-20a-5p, miR-340-5p, and let-7i-5p. (a) The temporal expression of miR-20a-5p, miR-340-5p, and let-7i-5p in the PBMCs of post-MI rats and sham controls at 4, 8, and 12 weeks post-MI. (b) Transmission electron microscope image of exosomes isolated from plasma (A; white arrow) and western blot of exosomal markers ALIX, TSG101, and CD63 (B). (c) Expression levels of exosome-original miR-20a-5p, miR-340-5p, and let-7i-5p at 8 weeks post-MI. ^∗^*P* < 0.05 versus the corresponding sham group; *^Φ^P* < 0.05 versus the corresponding sham group (miR-20a-5p); ^#^*P* < 0.05 versus the corresponding sham group (miR-340-5p); ^&^*P* < 0.05 versus the corresponding sham group (let-7i-5p). MI: myocardial infarction; PBMCs: peripheral blood mononuclear cells; ALIX: apoptosis-linked gene 2-interacting protein X; TSG101: tumour susceptibility gene 101 protein; CD63: cluster of differentiation 63.

We quantified the expression levels of miR-20a-5p, miR-340-5p, and let-7i-5p in the HF-related tissues, including the liver, spleen, lung, kidney, thymus, and PBMCs at 8 weeks post-MI. The expression levels of miR-20a-5p, miR-340-5p, and let-7i-5p were upregulated in PBMCs at 8 weeks post-MI (Figure 5(a)), and there was no significant difference in the miR-20a-5p, miR-340-5p, and let-7i-5p levels in the liver, spleen, lung, kidney, and thymus tissues between the 8-week post-MI group and the sham group (see [Supplementary-material supplementary-material-1] in the Supplementary Material for quantitative results). We subsequently investigated the temporal expression of miR-20a-5p, miR-340-5p, and let-7i-5p in PBMCs at 4 weeks and 12 weeks post-MI. The expression levels of miR-20a-5p and miR-340-5p in PBMCs showed an upward trend that was similar to the trend in plasma from 4 weeks to 8 weeks post-MI (Figure 5(a)). These results indicated that PBMCs may be a potential origin of plasma miR-20a-5p and miR-340-5p. Furthermore, exosomes were isolated from plasma (Figure 5(b)) and used to evaluate the expression levels of exosome-original miR-20a-5p, miR-340-5p, and let-7i-5p. Only the expression levels of miR-20a-5p showed a significant 1.7-fold increase in the 8-week post-MI group compared with the sham group, indicating that plasma miR-20a-5p may be partly transported by exosomes (Figure 5(c)).

### 3.6. Target mRNAs of miR-20a-5p, miR-340-5p, and let-7i-5p

**Figure 6:**
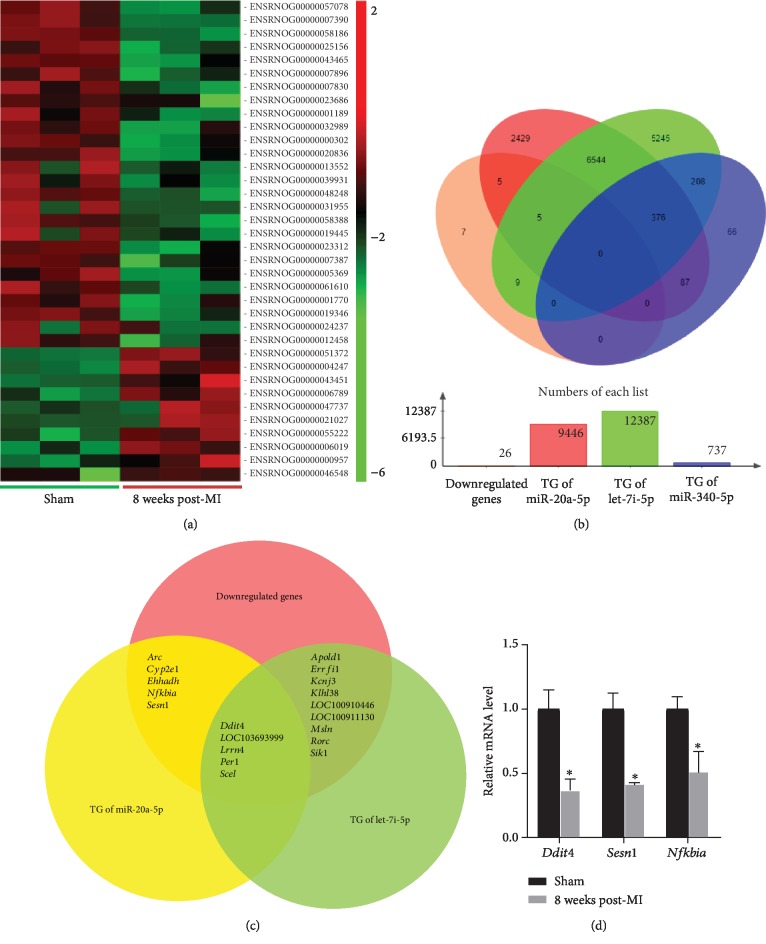
Target genes of plasma miR-20a-5p, miR-340-5p, and let-7i-5p. (a) Hierarchical clustering of 36 DE mRNAs (FC < 0.5 or >2; *P* < 0.05) in the 8-week post-MI group (*n* = 3) compared with the sham group (*n* = 3). (b) The number and overlap among the downregulated mRNAs generated from mRNA sequencing and the predicted target genes of miR-20a-5p, miR-340-5p, and let-7i-5p. (c) The common genes among the downregulated genes, the target genes of miR-20a-5p, and the target genes of let-7i-5p. (d) The expression levels of *Ddit4*, *Sesn1*, and *Nfkbia* mRNAs in the validated 8-week post-MI group (*n* = 4) compared with the corresponding sham group (*n* = 4), which were examined with real-time PCR. ^∗^*P* < 0.05 versus the sham group. DE: differentially expressed; mRNA: messenger RNA; MI: myocardial infarction; FC: fold change; TG: target genes; *Ddit4*: DNA-damage-inducible transcript 4; *Sesn1*: sestrin 1; *Nfkbia*: NF-*κ*B inhibitor *α*.

Plasma miR-20a-5p, miR-340-5p, and let-7i-5p may play important roles in the development of left ventricular remodeling. Since miRNAs are known to regulate gene expression via the degradation or translational repression of their target mRNAs [5], a parallel miRNA-mRNA expression profiling approach [23, 24] was used to identify potential mRNA targets of miR-20a-5p, miR-340-5p, and let-7i-5p. The global mRNA expression in the myocardium (an area distant from the site of infarction) of rats in the 8-week post-MI group and the corresponding sham group was analysed using mRNA sequencing. Differential expression analysis indicated that 36 mRNAs were differentially expressed in the 8-week post-MI group compared with the sham group (FC < 0.5 or >2; *P* < 0.05). Of the 36 differentially expressed mRNAs, 10 were upregulated and 26 were downregulated in the 8-week post-MI group compared with the sham group. Differentially expressed mRNAs clearly distinguished the 8-week post-MI group from the sham group (Figure 6(a)). The target genes of miR-20a-5p, miR-340-5p, and let-7i-5p were predicted using miRWalk, miRanda, RNAhybrid, and TargetScan analyses and subsequently paired to the downregulated mRNAs generated from mRNA sequencing (Figure 6(b)). The symbols of paired genes are presented in Figure 6(c) (19 genes in total). There was no paired gene between the targets of miR-340-5p and the downregulated myocardial mRNAs. However, there were 671 target genes of miR-340-5p in common with the target genes of miR-20a-5p and/or let-7i-5p. Additionally, several pathways mechanistically associated with cardiac remodeling were presented in the KEGG annotation results of the 19 common target genes, including the mechanistic target of rapamycin (mTOR), nuclear factor- (NF-) *κ*B, tumour necrosis factor (TNF), apoptosis, p53, and other signaling pathways (see [Supplementary-material supplementary-material-1] in Supplementary Materials for comprehensive KEGG analysis). From the 19 common genes, *Ddit4*, *Sesn1*, and *Nfkbia* were selected for further validation with real-time PCR in the 8-week post-MI group and the sham group. These 3 genes play roles in the cellular processes, including cellular growth, autophagy, apoptosis, inflammation, and fibrosis [25–29], which are associated with the progression of cardiac remodeling [30]. As presented in Figure 6(d), the expression levels of *Ddit4*, *Sesn1*, and *Nfkbia* mRNAs were also significantly reduced in the validated 8-week post-MI group compared with the corresponding sham group. *Ddit4*, *Sesn1*, and *Nfkbia* may be potential target genes of miR-20a-5p, and *Ddit4* may be a potential target gene of let-7i-5p.

### 3.7. Evaluation of Candidate Plasma miRNAs as Biomarkers for Postinfarction Left Ventricular Remodeling in Patient Population

**Figure 7:**
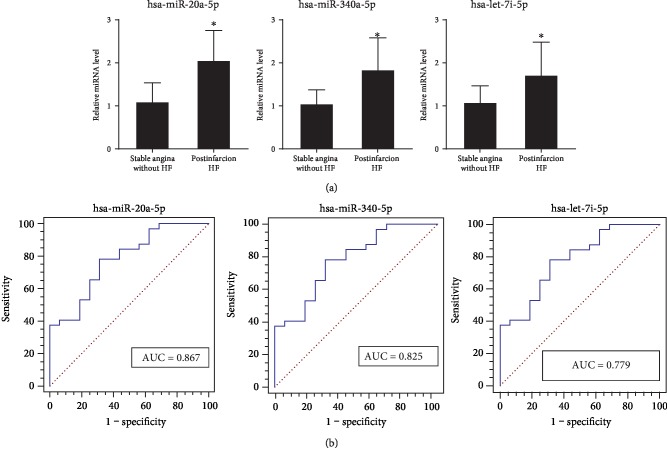
Expression of candidate plasma miRNAs in the patient population. (a) The expression levels of plasma miR-20a-5p, miR-340-5p, and let-7i-5p in patients with postinfarction HF (*n* = 32) and control patients with stable angina and without significant coronary lesions and HF (*n* = 16). (b) ROC curves and the AUCs of miR-20a-5p, miR-340-5p, and let-7i-5p. ^∗^*P* < 0.05 versus patients with stable angina and without significant coronary lesions and HF. HF: heart failure; ROC: receiver operator characteristic; AUC: area under the curve.

The sequences of miR-20a-5p, miR-340-5p, and let-7i-5p share significant homology between rats and humans. The expression levels of these 3 miRNAs were subsequently investigated in 32 patients with postinfarction HF and 16 control patients with stable angina and without significant coronary lesions and HF. The baseline characteristics of the subjects are presented in Table 2. The echocardiographic ejection fraction measurements of patients with postinfarction HF were lower than 60% and higher than 21%. The levels of NT-proBNP in patients with postinfarction HF were higher than 125 pg/ml. Additionally, 56.3% and 62.5% patients with postinfarction HF used *β*-receptor blockers and angiotensin-converting enzyme inhibitors/angiotensin receptor blockers, respectively, to treat postinfarction left ventricular remodeling and/or hypertension. No significant difference in the echocardiographic measurements of the interventricular septum and left ventricular posterior wall was identified between patients with postinfarction HF and controls. The plasma levels of miR-20a-5p, miR-340-5p, and let-7i-5p were significantly increased in patients with postinfarction HF compared with controls (Figure 7(a)). Additionally, the AUCs of miR-20a-5p, miR-340-5p, and let-7i-5p were 0.867 (*P* < 0.05), 0.825 (*P* < 0.05), and 0.779 (*P* < 0.05), respectively (Figure 7(b)). However, only the expression levels of miR-20a-5p presented significant positive correlations with LVIDd (Pearson correlation coefficient: 0.435; *P* value: 0.002) and left ventricular end diastolic volume (LVEDV; Pearson correlation coefficient: 0.323; *P* value: 0.025) (Table 3).

## 4. Discussion

Following MI, progressive cardiac remodeling is associated with cardiac dysfunction and poor prognosis for survival [31]. During the development of left ventricular remodeling, gross pathologic changes in the left ventricular configuration and volume evaluated by echocardiography have been widely used to predict the clinical outcomes of individuals [2]. However, on a molecular level, biomarkers for left ventricular remodeling are limited to proteins that reflect haemodynamic stress (such as natriuretic peptides) or necrosis (such as cardiac troponins) [12]. Recently, miRNAs, a class of evolutionarily conserved small noncoding RNAs, have been discovered to be stably transported in the blood [10]. Since miRNAs are known to function as gene expression repressors at the posttranscriptional level by targeting the corresponding mRNAs and play important roles in the development of HF, circulating miRNAs have been proposed to be promising biomarkers for left ventricular remodeling [5, 32, 33]. However, the temporal and tissue-specific expression of miRNAs following MI may affect the diagnostic specificity and reproducibility of the use of circulating miRNAs as biomarkers for left ventricular remodeling [11, 34]. In the current study, using a postinfarction HF rat cohort, our data revealed that the expression levels of plasma miR-20a-5p, miR-340-5p, and let-7i-5p gradually increased and were closely associated with left ventricular hypertrophy and dilatation from 4 weeks to 8 weeks post-MI. At 12 weeks post-MI, the mechanisms underlying left ventricular remodeling became maladaptive and led to significant deterioration in cardiac function and further left ventricular dilatation and thinning. The expression levels of plasma miR-340-5p and let-7i-5p were restored to baseline levels, and the expression levels of miR-20a-5p presented significant inverse correlations with the dilatation of the left ventricle at 12 weeks post-MI. The target genes of miR-20a-5p and let-7i-5p were involved in several pathways associated with the development of left ventricular remodeling. The expression levels of miR-20a-5p, miR-340-5p, and let-7i-5p were also temporally regulated in the myocardium and PBMCs. It may be suggested that these 3 miRNAs played functional roles in the pathophysiology of left ventricular remodeling, and the loss of increased miR-20a-5p, miR-340-5p, and let-7i-5p was associated with the process of maladaptive left ventricular remodeling. In the clinical study, patients with postinfarction HF presented a wide range of echocardiographic left ventricular phenotypes, and a lack of extreme phenotypes associated with severe left ventricular remodeling, which may be due to the development of left ventricular remodeling, can be affected by different infarct sizes and time points, antiremodeling medical therapy, and myocardial revascularization strategies. Similar to the expression pattern in postinfarction HF rats at 4 and 8 weeks post-MI, the expression levels of plasma miR-20a-5p, miR-340-5p, and let-7i-5p were increased in patients with postinfarction HF. Additionally, the expression levels of miR-20a-5p presented significant correlations with LVIDd and LVEDV. In accordance with our findings, in a community-based observational cohort, the expression levels of miR-20a-5p were demonstrated to be associated with the development of left ventricular remodeling and HF incidence during the follow-up period [35]. Therefore, miR-20a-5p may be a novel potential biomarker for postinfarction left ventricular remodeling.

The results in the present study indicated that miR-20a-5p and let-7i-5p targeted a set of genes involved in the pathways potentially associated with the pathophysiology of left ventricular remodeling, including the mTOR, NF-*κ*B, TNF, apoptosis, and p53 signaling pathways. Among these target genes, the expression levels of *Ddit4*, *Sesn1*, and *Nfkbia* mRNAs, which are closely associated with cellular growth, inflammation, and cell survival, were verified to be reduced in the myocardium of rats at 8 weeks post-MI. DDIT4 is a stress-induced protein and functions as an inhibitor of mTOR kinase [25, 36, 37]. mTOR kinase is an important regulator that promotes cellular growth and proliferation [38, 39]. *Sesn1* is a target gene of the tumour suppressor p53, a stress-activated transcription factor that inhibits cellular growth and proliferation [40]. The protein of *Nfkbia* is an inhibitor of NF-*κ*B transcription factor, which plays multifaceted roles in the regulation of inflammation and cell survival during the development of cardiac remodeling [41]. Additionally, previous studies supported the progrowth and antiapoptosis effects of miR-20a-5p and let-7i-5p on cardiomyocytes or myoblasts [42–45]. Therefore, *Ddit4*, *Sesn1*, and *Nfkbia* may be potential target genes of miR-20a-5p and/or let-7i-5p.

In patients with acute decompensated HF, the expression levels of plasma let-7i-5p were lower in patients with worsening of renal function compared with patients without worsening of renal function [46]. In a cohort of HF patients with and without atherosclerotic disease, the lower levels of plasma let-7i-5p were associated with the risk of rehospitalizations due to cardiovascular causes during 18 months of follow-up [45]. In the current study, the loss of increased let-7i-5p was associated with the phenotypes of adverse left ventricular remodeling. It may be suggested that let-7i-5p plays protective roles in the development of HF and the lower circulating let-7i-5p levels are associated with the poor prognosis of HF.

In the present study, our data indicated that the expression of miRNAs in plasma, myocardium, and PBMCs may be temporally interconnected. Similarly, in patients with dilated cardiomyopathy, the miRNA expression profile was specifically modulated in PBMCs, and many differentially expressed miRNAs were deregulated in the myocardium in previous studies [47]. Additionally, in an ischemia reperfusion mouse cohort, the expression levels of the majority of candidate miRNAs associated with left ventricular remodeling were upregulated in the myocardium at acute time points and in plasma at chronic time points [11]. However, these findings are exploratory and require further investigation in the *in vivo* and *in vitro* mechanistic studies.

## 5. Conclusions

Left ventricular remodeling is identified as a central pathophysiologic mechanism involved in the development of HF. Finding reliable biomarkers for left ventricular remodeling may facilitate the risk stratification and prognosis assessment of patients with HF. The results of the present study suggested that the expression levels of plasma miR-20a-5p, miR-340-5p, and let-7i-5p were associated with the development of left ventricular remodeling. The expression levels of plasma miR-20a-5p presented significant correlations with left ventricular dilatation in rats and patients with postinfarction HF. Therefore, miR-20a-5p may serve as a novel potential biomarker for cardiac remodeling during the development of postinfarction HF.

## Figures and Tables

**Table 1:** Correlations between the expression levels of plasma miRNAs and LVIDd, LVIDs, and cardiomyocyte size at 4 weeks, 8 weeks, and 12 weeks post-MI.

	LVIDd			LVIDs			Cardiomyocyte size		
	4 weeks	8 weeks	12 weeks	4 weeks	8 weeks	12 weeks	4 weeks	8 weeks	12 weeks
miR-20a-5p	*r* = 0.793^∗^ *P* = 0.019	*r* = 0.889^∗^ *P* = 0.003	*r* = −0.816^∗^ *P* = 0.013	*r* = 0.783^∗^ *P* = 0.022	*r* = 0.861^∗^ *P* = 0.006	*r* = −0.735^∗^ *P* = 0.038	*r* = 0.977^∗^ *P* = 0	*r* = 0.932^∗^ *P* = 0.01	*r* = −0.609 *P* = 0.109
miR-340-5p	*r* = 0.863^∗^ *P* = 0.006	*r* = 0.733^∗^ *P* = 0.039	*r* = −0.393 *P* = 0.336	*r* = 0.855^∗^ *P* = 0.007	*r* = 0.707^∗^ *P* = 0.049	*r* = −0.434 *P* = 0.283	*r* = 0.848^∗^ *P* = 0.008	*r* = 0.74^∗^ *P* = 0.036	*r* = −0.386 *P* = 0.345
let-7i-5p	*r* = 0.754^∗^ *P* = 0.031	*r* = 0.829^∗^ *P* = 0.011	*r* = −0.061 *P* = 0.887	*r* = 0696^∗^ *P* = 0.019	*r* = 0.799^∗^ *P* = 0.017	*r* = −0.302 *P* = 0.468	*r* = 0.719^∗^ *P* = 0.044	*r* = 0.868^∗^ *P* = 0.005	*r* = −0.009 *P* = 0.984

MI: myocardial infarction; *r*: Pearson correlation coefficient; LVIDd: left ventricular internal end diastolic dimension; LVIDs: left ventricular internal end systolic dimension; MI: myocardial infarction. ^∗^*P* < 0.05 versus the corresponding sham group.

**Table 2:** Baseline characteristics of the subjects.

Variable	Control (*n* = 16)	Postinfarction HF (*n* = 32)
Age (years)^Ɨ^	61.1 (12.4)	62.4 (10.2)
Female^¶^	9 (56.3)	17 (53.1)
Diabetes^¶^	4 (25.0)	10 (31.3)
Hypertension^¶^	8 (50.0)	15 (46.9)
Current smoker^¶^	3 (18.8)	5 (15.6)
Triglycerides (mmol/l)^Ɨ^	1.92 (1.62)	1.58 (1.02)
Total cholesterol (mmol/l)^Ɨ^	4.47 (1.81)	4.00 (1.00)
LDL (mmol/l)^Ɨ^	2.65 (1.13)	2.54 (0.89)
HDL (mmol/l)^Ɨ^	1.20 (0.26)	1.05 (0.21)
Creatinine (*μ*mol/l)^Ɨ^	73.47 (11.20)	84.47 (14.82)
*β*-Receptor blockers^¶^	4 (25.0)	18 (56.3)^∗^
ACE inhibitors or AR blockers^¶^	5 (31.3)	20 (62.5)^∗^
NT-proBNP (pg/ml)^§^	99.1 (71.5, 132.8)	831.0 (152.5, 1605.0)^∗^
Echocardiography		
LVIDd (mm)^Ɨ^	42.6 (2.3)	54.1 (7.7)^∗^
LVEDV (ml)^Ɨ^	105.4 (34.6)	159.9 (65.4)^∗^
Ejection fraction (%)^Ɨ^	73.2 (3.7)	44.5 (9.4)^∗^
Fractional shortening (%)^Ɨ^	42.0 (2.8)	21.3 (5.6)^∗^
IVS (mm)^Ɨ^	9.5 (1.9)	10.3 (1.6)
LVPW (mm)^Ɨ^	9.5 (1.8)	9.8 (1.2)

^Ɨ^Mean (SEM), no. (%), and ^§^median (25% percentile, 75% percentile) of nonnormally distributed variables. HF: heart failure; LDL: low-density lipoprotein cholesterol; HDL: high-density lipoprotein cholesterol; ACE: angiotensin-converting enzyme; AR: angiotensin receptor; NT-proBNP: N-terminal pro-B type natriuretic peptide; LVIDd: left ventricular internal end diastolic dimension; LVEDV: left ventricular end diastolic volume; IVS: interventricular septum; LVPW: left ventricular posterior wall. ^∗^*P* < 0.05 versus controls.

**Table 3:** Correlations between the expression levels of plasma miRNAs and LVIDd, LVEDV, IVS, and LVPW in the patient population.

	LVIDd	LVEDV	IVS	LVPW
hsa-miR-20a-5p	*r* = 0.435^∗^, *P* = 0.002	*r* = 0.323^∗^, *P* = 0.025	*r* = 0.218, *P* = 0.137	*r* = 0.199, *P* = 0.174
hsa-miR-340-5p	*r* = 0.219, *P* = 0.134	*r* = 0.225, *P* = 0.124	*r* = 0.180, *P* = 0.221	*r* = 0.018, *P* = 0.904
hsa-let-7i-5p	*r* = 0.200, *P* = 0.173	*r* = 0.223, *P* = 0.128	*r* = 0.062, *P* = 0.675	*r* = 0.090, *P* = 0.544

HF: heart failure; *r*: Pearson correlation coefficient; LVIDd: left ventricular internal end diastolic dimension; LVEDV: left ventricular end diastolic volume; IVS: interventricular septum; LVPW: left ventricular posterior wall. ^∗^*P* < 0.05 versus stable angina patients without significant coronary lesions and HF.

## Data Availability

The database used to support the findings of this study is available from the corresponding author upon request.

## Supplementary Materials

Supplementary Materials Supplementary Figure 1: expression levels of miR-20a-5p, miR-340-5p, and let-7i-5p in the liver, spleen, lung, kidney, and thymus tissues in the 8-week post-MI group and the sham group. Supplementary Table 1: the KEGG annotation results of the 19 common target genes.
