# Corrigendum to “Circulating microRNAs as Novel Potential Biomarkers for Left Ventricular Remodeling in Postinfarction Heart Failure”

**DOI:** 10.1155/2022/9781913

**Published:** 2022-05-17

**Authors:** Guangyuan Gao, Weiwei Chen, Miao Liu, Xu Yan, Ping Yang

**Affiliations:** ^1^Department of Cardiology, China-Japan Union Hospital of Jilin University, Changchun 130031, China; ^2^Jilin Provincial Molecular Biology Research Center for Precision Medicine of Major Cardiovascular Disease, Changchun 130031, China

In the article titled “Circulating MicroRNAs as Novel Potential Biomarkers for Left Ventricular Remodeling in Postinfarction Heart Failure” [1], a duplication of the panels in Figure 7(b) was noted on PubPeer [2].

The authors have re-examined the data and explained that the error occurred due to the incorrect use of the ROC curve drawing software (MedCale). The authors provided the corrected ROC curve graphs and the underlying data. The corrected Figure 7(b) is shown below:

## Figures and Tables

**Figure 1:**
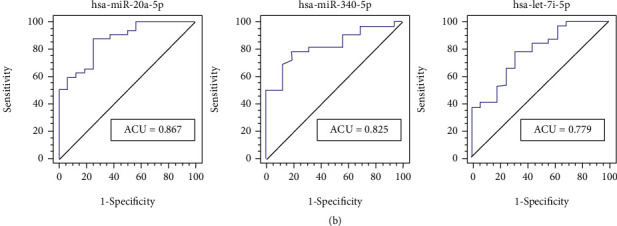
Expression of candidate plasma miRNAs in the patient population. (a) The expression levels of plasma miR-20a-5p, miR-340-5p, and let-7i-5p in patients with postinfarction HF (*n* = 32) and control patients with stable angina and without significant coronary lesions and HF (*n* = 16). (b) ROC curves and the AUCs of miR-20a-5p, miR-340-5p, and let-7i-5p. ^∗^*P* < 0 : 05 versus patients with stable angina and without significant coronary lesions and HF. HF: heart failure; ROC: receiver operator characteristic; AUC: area under the curve.
