# Supplementary material for: Circulating MicroRNAs as Novel Potential Biomarkers for Left Ventricular Remodeling in Postinfarction Heart Failure
Source: Dis Markers. 2019 Dec 2;2019:5093803. doi: 10.1155/2019/5093803 (PMC6914954; doi:10.1155/2019/5093803)
Supplement: Supplementary Materials — Supplementary Figure 1: expression levels of miR-20a-5p, miR-340-5p, and let-7i-5p in the liver, spleen, lung, kidney, and thymus tissues in the 8-week post-MI group and the sham group. Supplementary Table 1: the KEGG annotation results of the 19 common target genes. [file 5093803.f1.pdf]

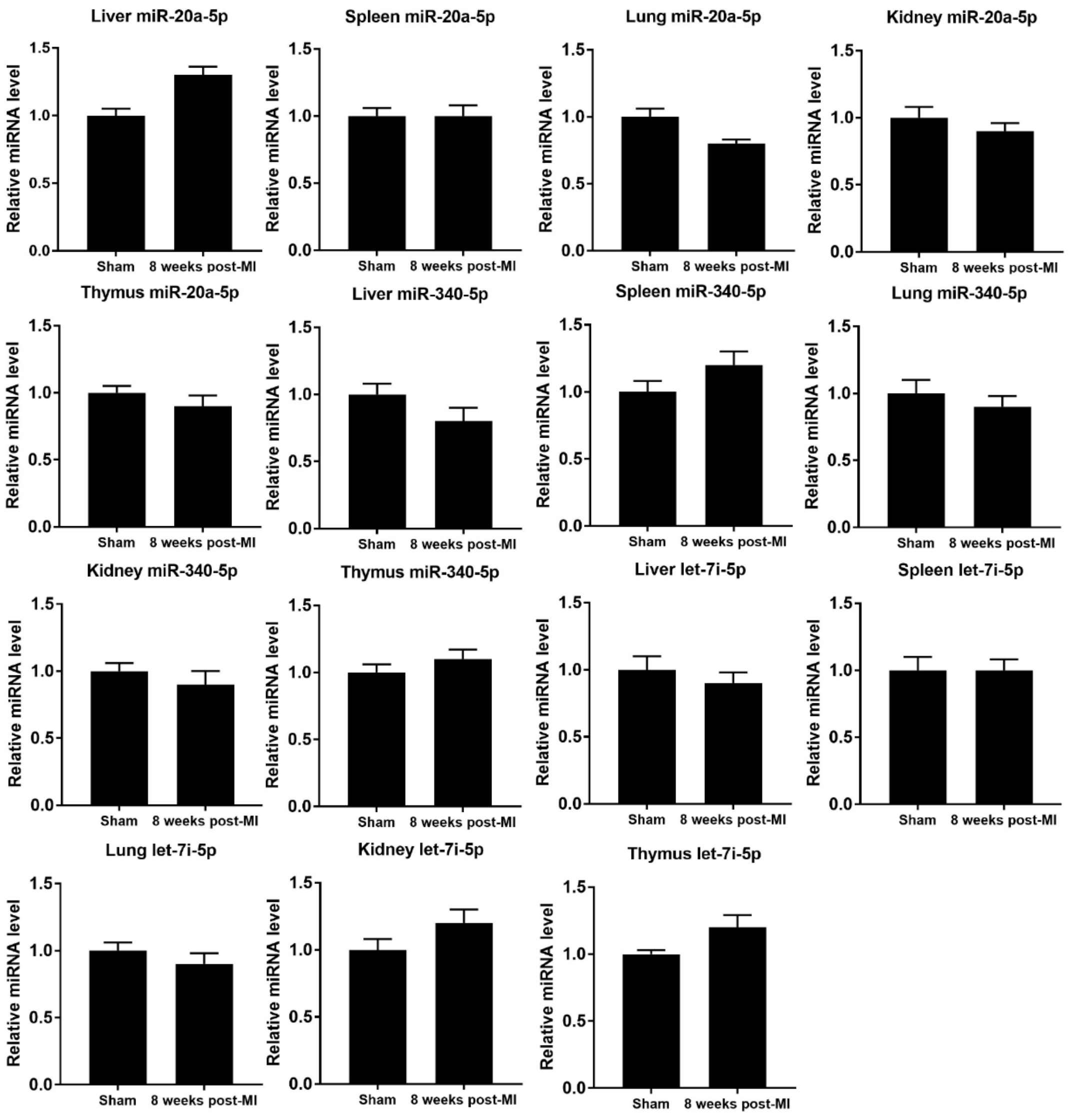

Figure S1: Expression levels of miR-20a-5p, miR-340-5p, and let-7i-5p in liver, spleen, lung, kidney and thymus tissues in the 8 weeks post-MI group and the sham group. There was no significant difference in the miR-20a-5p, miR-340-5p, and let-7i-5p levels in liver, spleen, lung, kidney and thymus tissues between the 8 weeks post-MI group and the sham group. MI, myocardial infarction.

Table S1: The KEGG annotation results of the 19 common target genes.

Pathway	ID	Input	Hyperlink
Circadian rhythm	rno04710	ENSRNOG00000007387|ENSRNOG00000020836	http://www.genome.jp/kegg-bin/show_pathway?rno04710/rno:287422%09red/rno:368158%09red
Circadian entrainment	rno04713	ENSRNOG00000007387|ENSRNOG00000005369	http://www.genome.jp/kegg-bin/show_pathway?rno04713/rno:50599%09red/rno:287422%09red
Herpes simplex infection	rno05168	ENSRNOG00000007387|ENSRNOG00000007390	http://www.genome.jp/kegg-bin/show_pathway?rno05168/rno:287422%09red/rno:25493%09red
Butanoate metabolism	rno00650	ENSRNOG00000001770	http://www.genome.jp/kegg-bin/show_pathway?rno00650/rno:171142%09red
beta-Alanine metabolism	rno00410	ENSRNOG00000001770	http://www.genome.jp/kegg-bin/show_pathway?rno00410/rno:171142%09red
Propanoate metabolism	rno00640	ENSRNOG00000001770	http://www.genome.jp/kegg-bin/show_pathway?rno00640/rno:171142%09red
Linoleic acid metabolism	rno00591	ENSRNOG00000012458	http://www.genome.jp/kegg-bin/show_pathway?rno00591/rno:25086%09red
Tryptophan metabolism	rno00380	ENSRNOG00000001770	http://www.genome.jp/kegg-bin/show_pathway?rno00380/rno:171142%09red
Fatty acid degradation	rno00071	ENSRNOG00000001770	http://www.genome.jp/kegg-bin/show_pathway?rno00071/rno:171142%09red
Lysine degradation	rno00310	ENSRNOG00000001770	http://www.genome.jp/kegg-bin/show_pathway?rno00310/rno:171142%09red
Fatty acid metabolism	rno01212	ENSRNOG00000001770	http://www.genome.jp/kegg-bin/show_pathway?rno01212/rno:171142%09red
Valine, leucine and isoleucine degradation	rno00280	ENSRNOG00000001770	http://www.genome.jp/kegg-bin/show_pathway?rno00280/rno:171142%09red
Cytosolic DNA-sensing pathway	rno04623	ENSRNOG00000007390	http://www.genome.jp/kegg-bin/show_pathway?rno04623/rno:25493%09red
Legionellosis	rno05134	ENSRNOG00000007390	http://www.genome.jp/kegg-bin/show_pathway?rno05134/rno:25493%09red
NOD-like receptor signaling pathway	rno04621	ENSRNOG00000007390	http://www.genome.jp/kegg-bin/show_pathway?rno04621/rno:25493%09red
RIG-I-like receptor signaling pathway	rno04622	ENSRNOG00000007390	http://www.genome.jp/kegg-bin/show_pathway?rno04622/rno:25493%09red
Amphetamine addiction	rno05031	ENSRNOG00000043465	http://www.genome.jp/kegg-bin/show_pathway?rno05031/rno:54323%09red

Inflammatory bowel disease (IBD)	rno05321	ENSRNOG00000020836	http://www.genome.jp/kegg-bin/show_pathway?rno05321/rno:368158%09red
Metabolism of xenobiotics by cytochrome P450	rno00980	ENSRNOG00000012458	http://www.genome.jp/kegg-bin/show_pathway?rno00980/rno:25086%09red
p53 signaling pathway	rno04115	ENSRNOG00000000302	http://www.genome.jp/kegg-bin/show_pathway?rno04115/rno:294518%09red
Drug metabolism - cytochrome P450	rno00982	ENSRNOG00000012458	http://www.genome.jp/kegg-bin/show_pathway?rno00982/rno:25086%09red
Steroid hormone biosynthesis	rno00140	ENSRNOG00000012458	http://www.genome.jp/kegg-bin/show_pathway?rno00140/rno:25086%09red
Leishmaniasis	rno05140	ENSRNOG00000007390	http://www.genome.jp/kegg-bin/show_pathway?rno05140/rno:25493%09red
Arachidonic acid metabolism	rno00590	ENSRNOG00000012458	http://www.genome.jp/kegg-bin/show_pathway?rno00590/rno:25086%09red
B cell receptor signaling pathway	rno04662	ENSRNOG00000007390	http://www.genome.jp/kegg-bin/show_pathway?rno04662/rno:25493%09red
Adipocytokine signaling pathway	rno04920	ENSRNOG00000007390	http://www.genome.jp/kegg-bin/show_pathway?rno04920/rno:25493%09red
Chronic myeloid leukemia	rno05220	ENSRNOG00000007390	http://www.genome.jp/kegg-bin/show_pathway?rno05220/rno:25493%09red
PPAR signaling pathway	rno03320	ENSRNOG00000001770	http://www.genome.jp/kegg-bin/show_pathway?rno03320/rno:171142%09red
Chemical carcinogenesis	rno05204	ENSRNOG00000012458	http://www.genome.jp/kegg-bin/show_pathway?rno05204/rno:25086%09red
Small cell lung cancer	rno05222	ENSRNOG00000007390	http://www.genome.jp/kegg-bin/show_pathway?rno05222/rno:25493%09red
Glucagon signaling pathway	rno04922	ENSRNOG00000001189	http://www.genome.jp/kegg-bin/show_pathway?rno04922/rno:59329%09red
Prostate cancer	rno05215	ENSRNOG00000007390	http://www.genome.jp/kegg-bin/show_pathway?rno05215/rno:25493%09red
Peroxisome	rno04146	ENSRNOG00000001770	http://www.genome.jp/kegg-bin/show_pathway?rno04146/rno:171142%09red
NF-kappa B signaling pathway	rno04064	ENSRNOG00000007390	http://www.genome.jp/kegg-bin/show_pathway?rno04064/rno:25493%09red

Longevity regulating pathway	rno04211	ENSRNOG00000000302	http://www.genome.jp/kegg-bin/show_pathway?rno04211/rno:294518%09red
Morphine addiction	rno05032	ENSRNOG00000005369	http://www.genome.jp/kegg-bin/show_pathway?rno05032/rno:50599%09red
Estrogen signaling pathway	rno04915	ENSRNOG00000005369	http://www.genome.jp/kegg-bin/show_pathway?rno04915/rno:50599%09red
Toll-like receptor signaling pathway	rno04620	ENSRNOG00000007390	http://www.genome.jp/kegg-bin/show_pathway?rno04620/rno:25493%09red
Retrograde endocannabinoid signaling	rno04723	ENSRNOG00000005369	http://www.genome.jp/kegg-bin/show_pathway?rno04723/rno:50599%09red
Chagas disease (American trypanosomiasis)	rno05142	ENSRNOG00000007390	http://www.genome.jp/kegg-bin/show_pathway?rno05142/rno:25493%09red
T cell receptor signaling pathway	rno04660	ENSRNOG00000007390	http://www.genome.jp/kegg-bin/show_pathway?rno04660/rno:25493%09red
Insulin resistance	rno04931	ENSRNOG00000007390	http://www.genome.jp/kegg-bin/show_pathway?rno04931/rno:25493%09red
Cholinergic synapse	rno04725	ENSRNOG00000005369	http://www.genome.jp/kegg-bin/show_pathway?rno04725/rno:50599%09red
TNF signaling pathway	rno04668	ENSRNOG00000007390	http://www.genome.jp/kegg-bin/show_pathway?rno04668/rno:25493%09red
Glutamatergic synapse	rno04724	ENSRNOG00000005369	http://www.genome.jp/kegg-bin/show_pathway?rno04724/rno:50599%09red
Carbon metabolism	rno01200	ENSRNOG00000001770	http://www.genome.jp/kegg-bin/show_pathway?rno01200/rno:171142%09red
Toxoplasmosis	rno05145	ENSRNOG00000007390	http://www.genome.jp/kegg-bin/show_pathway?rno05145/rno:25493%09red
Serotonergic synapse	rno04726	ENSRNOG00000005369	http://www.genome.jp/kegg-bin/show_pathway?rno04726/rno:50599%09red
Neurotrophin signaling pathway	rno04722	ENSRNOG00000007390	http://www.genome.jp/kegg-bin/show_pathway?rno04722/rno:25493%09red
Dopaminergic synapse	rno04728	ENSRNOG00000005369	http://www.genome.jp/kegg-bin/show_pathway?rno04728/rno:50599%09red
Hepatitis C	rno05160	ENSRNOG00000007390	http://www.genome.jp/kegg-bin/show_pathway?rno05160/rno:25493%09red
Measles	rno05162	ENSRNOG00000007390	http://www.genome.jp/kegg-bin/show_pathway?rno05162/rno:25493%09red
Osteoclast differentiation	rno04380	ENSRNOG00000007390	http://www.genome.jp/kegg-bin/show_pathway?rno04380/rno:25493%09red

Apoptosis	rno04210	ENSRNOG00000007390	http://www.genome.jp/kegg-bin/show_pathway?rno04210/rno:25493%09red
Hepatitis B	rno05161	ENSRNOG00000007390	http://www.genome.jp/kegg-bin/show_pathway?rno05161/rno:25493%09red
Non-alcoholic fatty liver disease (NAFLD)	rno04932	ENSRNOG00000012458	http://www.genome.jp/kegg-bin/show_pathway?rno04932/rno:25086%09red
Oxytocin signaling pathway	rno04921	ENSRNOG00000005369	http://www.genome.jp/kegg-bin/show_pathway?rno04921/rno:50599%09red
mTOR signaling pathway	rno04150	ENSRNOG00000057078	http://www.genome.jp/kegg-bin/show_pathway?rno04150/rno:140942%09red
Influenza A	rno05164	ENSRNOG00000007390	http://www.genome.jp/kegg-bin/show_pathway?rno05164/rno:25493%09red
Chemokine signaling pathway	rno04062	ENSRNOG00000007390	http://www.genome.jp/kegg-bin/show_pathway?rno04062/rno:25493%09red
cAMP signaling pathway	rno04024	ENSRNOG00000007390	http://www.genome.jp/kegg-bin/show_pathway?rno04024/rno:25493%09red
Viral carcinogenesis	rno05203	ENSRNOG00000007390	http://www.genome.jp/kegg-bin/show_pathway?rno05203/rno:25493%09red
Epstein-Barr virus infection	rno05169	ENSRNOG00000007390	http://www.genome.jp/kegg-bin/show_pathway?rno05169/rno:25493%09red
MicroRNAs in cancer	rno05206	ENSRNOG00000057078	http://www.genome.jp/kegg-bin/show_pathway?rno05206/rno:140942%09red
HTLV-I infection	rno05166	ENSRNOG00000007390	http://www.genome.jp/kegg-bin/show_pathway?rno05166/rno:25493%09red
PI3K-Akt signaling pathway	rno04151	ENSRNOG00000057078	http://www.genome.jp/kegg-bin/show_pathway?rno04151/rno:140942%09red
Pathways in cancer	rno05200	ENSRNOG00000007390	http://www.genome.jp/kegg-bin/show_pathway?rno05200/rno:25493%09red
Metabolic pathways	rno01100	ENSRNOG00000012458|ENSRNOG00000001770	http://www.genome.jp/kegg-bin/show_pathway?rno01100/rno:171142%09red/rno:25086%09red
